# P-1191. A Phase 3b, Open-label, Single-arm Study in Adolescent and Adult Female Participants to Evaluate Clinical Symptom Improvement and Safety of Gepotidacin During Treatment of Uncomplicated Urinary Tract Infections (uUTIs)

**DOI:** 10.1093/ofid/ofaf695.1384

**Published:** 2026-01-11

**Authors:** Marcela Ramírez, Kalpana Gupta, Jazmín Díaz-Regañón, Rudrani Banerjee, Jagriti Bablani, Salim Janmohamed

**Affiliations:** GSK, London, UK, London, England, United Kingdom; Boston University School of Medicine, Boston, Massachusetts; GSK, Madrid, Spain, Madrid, Madrid, Spain; GSK, Bangalore, Karnataka, India; GSK, Bengaluru, India, Bengaluru, Karnataka, India; GSK, Brentford, UK, Brentford, England, United Kingdom

## Abstract

**Background:**

uUTIs can be painful and disrupt patients’ lives; thus, rapid symptom relief is a priority. Gepotidacin, a recently approved, novel, first-in-class triazaacenaphthylene oral antibacterial, was non-inferior to nitrofurantoin in two Phase 3 uUTI trials (EAGLE-2 [NCT04020341]/EAGLE-3 [NCT04187144]), and superior in EAGLE-3, with an acceptable safety profile; however, clinical response at 24h was not assessed.

**Figure:**
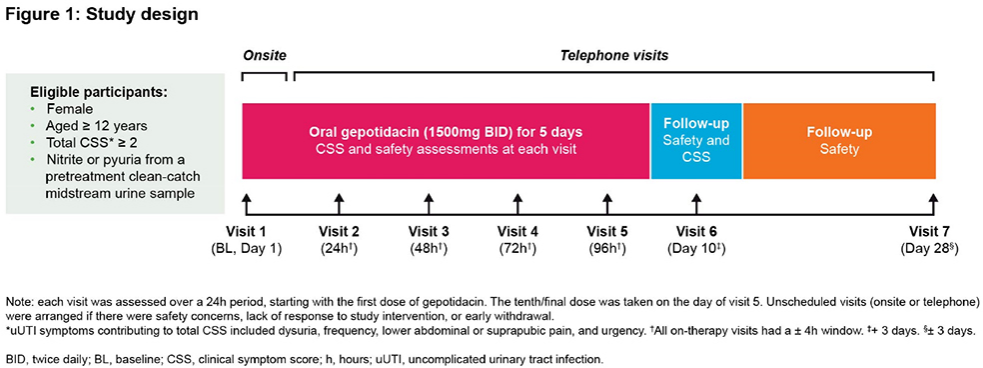

**Figure:**
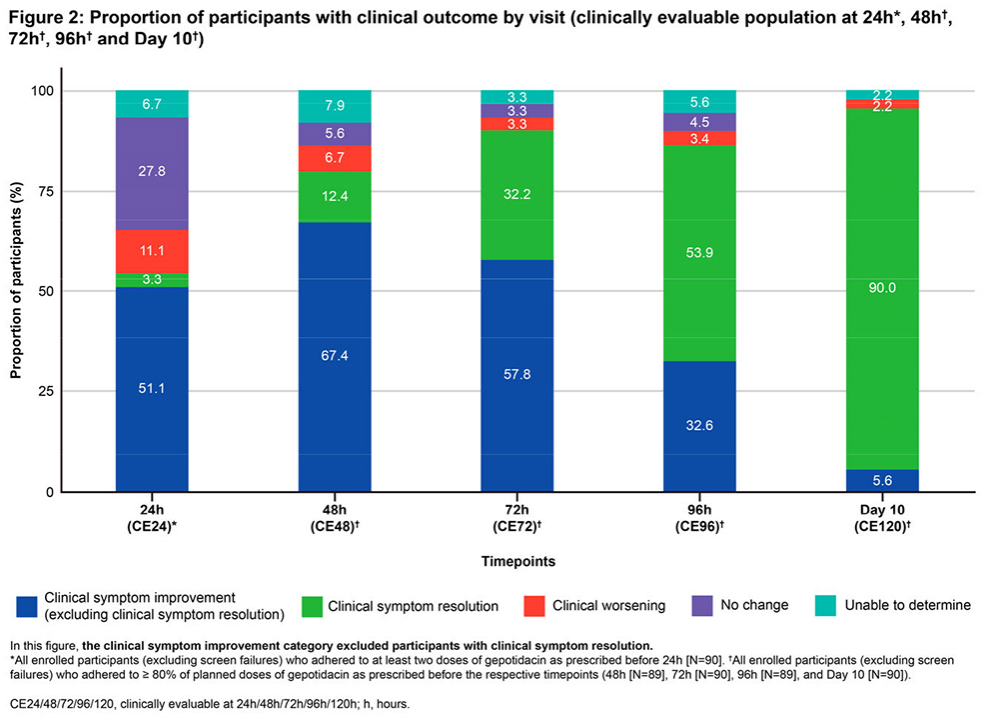

**Methods:**

This Phase 3b, open-label, single-arm, multicenter US study (NCT06597344) assessed uUTI symptom relief with oral gepotidacin (1500mg; twice daily for 5 days; Fig 1). Females aged ≥ 12 years with urinary nitrite or pyuria, and with ≥ 2 uUTI symptoms (listed in Fig 1) were eligible for the study. Each symptom was rated 0–3 (none–severe) and summed for a total clinical symptom score (CSS; range 0–12). Participants (pts) attended an in-person baseline (BL) visit (Day [D]1) and 6 telephone calls at 24h, 48h, 72h, 96h, D10 and D28 post BL. The primary objective was to assess clinical symptom improvement (CSI; decrease from BL in total CSS of ≥ 1 point, without requiring other antibacterials) with gepotidacin at 24h. Secondary and exploratory objectives were to assess CSI at 48h, 72h, 96h, and D10, clinical symptom resolution (CSR; total CSS decrease from BL to 0, without requiring other antibacterials) at 24h, 48h, 72h, 96h, and D10, and safety until D28.

**Figure:**
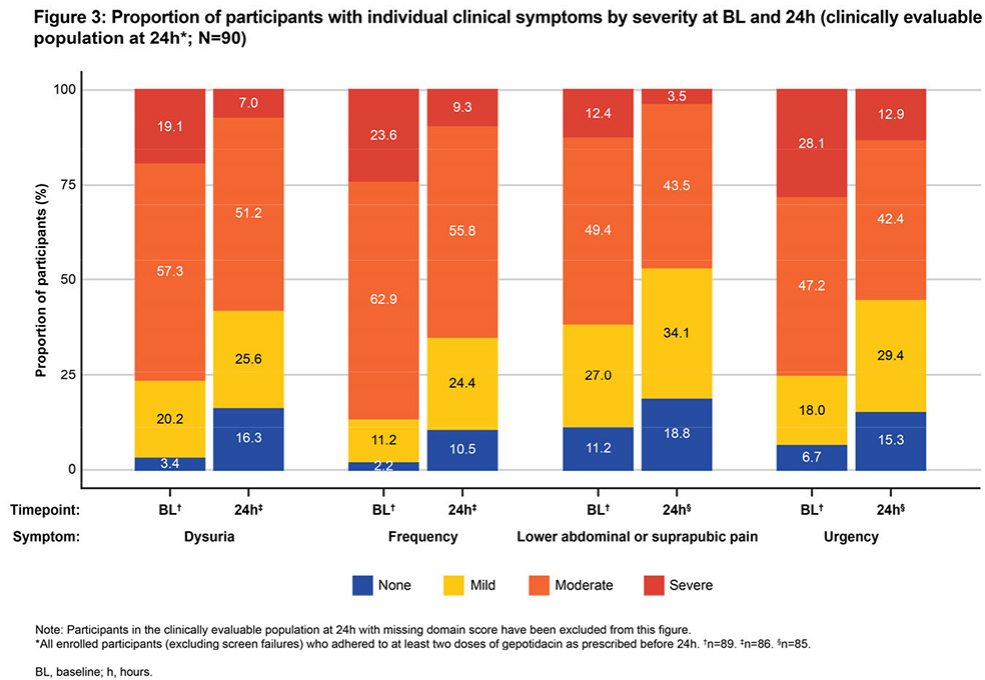

**Figure:**
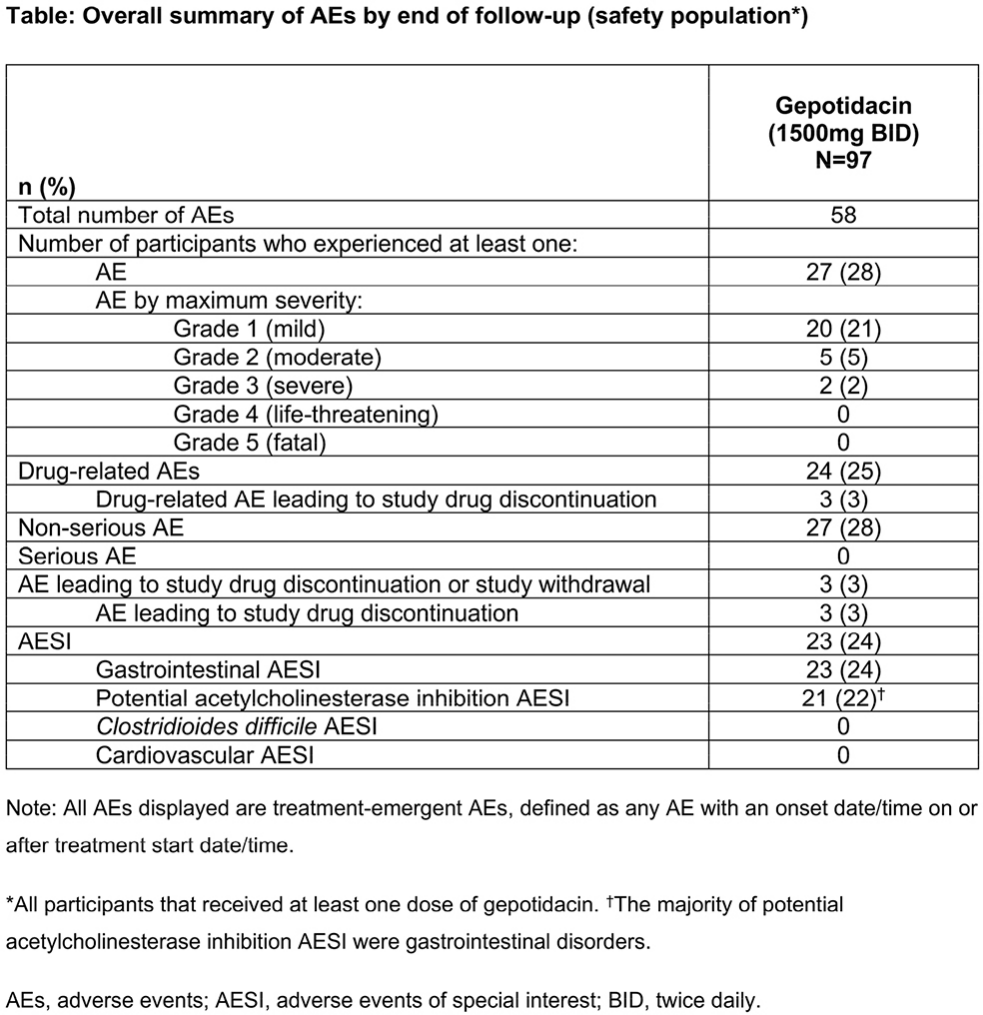

**Results:**

Of 97 pts enrolled, 90 were clinically evaluable (CE) at 24h (CE24 [primary analysis] population; defined in Fig 2). Mean (standard deviation) BL total CSS was 7.6 (2.11). In the CE24 population, 54.4% had CSI at 24h (Fig 2). Subsequently, 79.8% (CE48, N=89), 90.0% (CE72, N=90), and 86.5% (CE96, N=89) of CE pts had CSI at 48h, 72h, and 96h, respectively, increasing to 95.6% at D10 (CE120, N=90). CSR steadily increased to 90.0% at D10 (CE120). Severity of each individual symptom decreased by 24h (Fig 3). Adverse events (AEs) were mostly gastrointestinal and mild/moderate in severity; no pts had serious AEs (Table).

**Conclusion:**

Gepotidacin demonstrated a rapid and sustained clinical response, with > 50% of participants experiencing symptom improvement in 1 day of treatment and nearly 80% in 2 days. By Day 10, 90% of participants had achieved symptom resolution. An acceptable safety profile was reported.

Funding: GSK (study 219575).

**Disclosures:**

Marcela Ramírez, MD, GSK: Employee|GSK: Stocks/Bonds (Public Company) Kalpana Gupta, MD, GSK: Advisor/Consultant|Innotive Diagnostics: Advisor/Consultant|Iterum Therapeutics: Advisor/Consultant|Qiagen Inc.: Advisor/Consultant|UpToDate: Royalties|Utility Therapeutics: Advisor/Consultant Jazmín Díaz-Regañón, MD, GSK: Employee|GSK: Stocks/Bonds (Public Company) Rudrani Banerjee, PhD, GSK: Employee|GSK: Stocks/Bonds (Public Company) Jagriti Bablani, MSc, GSK: Employee Salim Janmohamed, MD, GSK: Employee|GSK: Stocks/Bonds (Public Company)

